# The Variety of Vertebrate Mechanisms of Sex Determination

**DOI:** 10.1155/2013/587460

**Published:** 2013-12-04

**Authors:** Antonina V. Trukhina, Natalia A. Lukina, Natalia D. Wackerow-Kouzova, Alexander F. Smirnov

**Affiliations:** Department of Genetics and Biotechnology, Saint-Petersburg State University, Saint-Petersburg 199034, Russia

## Abstract

The review deals with features of sex determination in vertebrates. The mechanisms of sex determination are compared between fishes, amphibians, reptilians, birds, and mammals. We focus on structural and functional differences in the role of sex-determining genes in different vertebrates. Special attention is paid to the role of estrogens in sex determination in nonmammalian vertebrates.

## 1. Peculiar Properties of Sex Determination among Different Vertebrates

One of the most fundamental and surprisingly diverse processes in the life history of an organism is the determination of sex. Its determination must be a very ancient process, with male and female sexes recognized in diverse organisms, from corals to worms, insects, fishes, birds, and mammals [1–4]. The transformation of three stages of sex formation (chromosomal predetermination, sex determination, and sex differentiation) was described in different groups of vertebrate. Sex determination at vertebrates has long been equated with gonadal differentiation into ovaries or testes. Consideration of different taxonomic animal groups allowed for establishing two general mechanisms of sex determination: genetic sex determination (GSD) and external sex determination (ESD). ESD at vertebrates is practically reduced to the temperature sex determination (TSD). Birds and mammals are characterized only by GSD, whereas crocodiles by TSD. Lizards, snakes, turtles, and bony fishes were described to have all possible mechanisms of sex determination [1]. There are two genetic sex determination systems: with heterogametic male—XY (mammals) and heterogametic females—ZW (birds). Note that both genetic systems are found in amphibians [1].

In organisms with heteromorphic sex chromosomes, such as birds and mammals, sex is set at fertilization by the differential inheritance of sex chromosomes [2–4]. The logical assumption is that sex-determining genes, inherited at fertilization, become active in the gonads during embryonic or larval life. However, the various reports of somatic sexual dimorphisms preceding the gonadal development call for a more considered definition of sex determination [4].

TSD was firstly discovered in reptiles: turtles, crocodiles, but not in snakes. The primary mechanism of sex determination is poorly understood. It obviously occurs in species with undifferentiated Y-chromosome. Higher temperature can produce either males or females, and the temperature ranges and lengths of exposure that influence TSD are remarkably variable among species. The classical view proposed that the developing gonads in vertebrate have the bipotential genital ridges: the cortex and the medulla. Thereafter, the process of sex differentiation depends on the alternative development of these two territories. Ovaries develop from the growing cortex, while testes develop from the medulla with an apparent antagonism between the two processes [5].

Although most genes involved in gonadal development are conserved at vertebrates, including species with TSD, the temporal and spatial gene expression patterns vary among species. Aromatase (CYP19), which regulates gonadal estrogen level, is proposed to be the main target of a putative thermosensitive factor for TSD. It is known that the estrogen levels may influence sex determination or gonad differentiation depending on the species. Yolk steroids of maternal origin and steroids produced by the embryonic nervous system should also be considered as sources of hormones that may play a role in TSD. It is an exception that different taxonomic groups of animals with TSD have different sex determination mechanisms. Moreover, there are thermosensitive genes: in* Emydidae—sox9*, in *Testudinae—sox9*, *sf1*, and *wt1*, and in *Emydidae—dax1*. It was proposed that in the case of temperature-dependent sex determination, a gene chain *amh*-*sox9 *operates affecting the appearance of testes in contrast to the other chain genes in mammalian sex determination *sry*-*sox9*-*amh* [5, 6].

Teleost fishes (over 30,000 species) are the largest group of vertebrates which exhibit a remarkable variety of sexuality. Fish sexualities were categorized into gonochorism, synchronous/sequential hermaphrodite, or unisexual reproduction. Sex at fishes is determined genetically or by environmental factors [7]. The only known exception of unisexual species is the amazon mollies—*Poecilia formosa.* Sequential hermaphrodite (sex-changing) species have been recorded in 27 of 448 families across 7 orders of fishes, most of which have found a niche in coral reefs. In these fishes, the gonadal sex redifferentiation was observed during sex change in adulthood. Thus, the sex-changing fishes are ideal models to investigate gonadal differentiation in vertebrates. Wrasses (*Labridae*) are the major group of species that display protogynous sex change. Individuals of this species initially mature as either males or females. Protandrous and protogynous sex changes are generally irreversible. Thus, such sex changes happen once in a life cycle. There are fish species with XX/XY and the most common ZW/ZZ sex determination system; the unusual WXZ system is described in swordtail (platyfish) [7]. Not many fish species have morphologically different sex chromosomes (10%) and most of them are at an early stage of differentiation. Even in the case of animals where sex is determined by genetic factors, the molecular processes that lead to the formation of either testis or ovary are evolutionary labile. The sex determination in most therian mammals is triggered by the testis-determining gene *sry.* This role is played by *dmy*/*dmrt1bY* and *dmrt1 *in medaka (*Oryzias latipes*) and chicken, respectively. Teleost fishes represent nearly half of all extant vertebrates and show a wide variety of sex determination mechanisms. Their sex can be determined by genetic factors or/and environmental factors. Recently, four novel sex determination (SD) genes or strong SD gene candidates in vertebrates were reported, all of them are in fishes: *amhy* in the Patagonian pejerrey *Odontesthes hatcheri*, *gsdf* in *Oryzias luzonensis* (a relative of medaka), *amhr2* in fugu *Takifugu rubripes*, and *sdY* in rainbow trout *Oncorhynchus mykiss* [8]. Complex epistatic sex system at fishes has been found consisting of a major female heterogametic ZW locus on chromosome 5, two separate male heterogametic XY loci on chromosome 7, and two additional interacting loci on chromosomes 3 and 20 [9].

Fishes have the most plastic system of germ and somatic cells in comparison with other animals. For them, the plasticity is maintained throughout the life cycle. It describes the impact on the process of such factors as temperature, pH, and population density. TSD at fish is less common than previously thought. The effect of estrogen acting through ER directly or indirectly regulates P450arom and AMH. The analysis of the differences between gonochoristic and hermaphroditic fish species will help to understand the mechanism of plasticity of sex determination in vertebrates [7].

Amphibians have two systems of sex chromosomes: one with heterogametic male (XX/XY) and another with the female (ZZ/ZW). Most urodeles (urodele salamanders) have XX/XY system. For 63 of 1500 species with determined sex, only 20 of the total number had differing sex chromosomes. There are heterogametic males in the ancestral species of toads *Leiopelma hamiltoni* and *L*. *hochstetteri*. Models of sex differentiation in amphibians can be divided into three types: (1) direct development of the undifferentiated gonad in testes or ovaries; (2) undifferentiated gonad development in the ovaries and testes through subsequent appearance of ovary; and (3) semidifferentiated called type-development phase of the testes of intersex. Most amphibians do not exhibit morphologically distinguishable sex chromosomes. In *Rana rugosa*, the X and W and also the Y and Z chromosomes are almost identical to each other based on the morphology and replication banding patterns, respectively. The Z shares its origin with the Y and the X—with the W. For a long time no sex-determining genes have been identified in amphibians. Recently [10, 11], a candidate for an ovary-determining gene, or *DM-W*, a W-linked DM-domain of gene was isolated from *Xenopus laevis. *However, the target gene downstream of *DM-W* is not known. To date, *DM-W* has not been found in any species of amphibian other than *X. laevis.* When *DM-W *is introduced into unfertilized eggs, ZZ transgenic male tadpoles form ovaries [10, 11].

*Dmrt1* gene is an autosomal gene in *X. laevis. *Moreover, there is the fact that the phenotypic sex of *X. laevis* carrying a pair of Z chromosomes can be altered from male to female by estrogens. It suggests that the mechanism of sex determination is flexible in this species. Rigid sex determination would need a prolonged expression of sex-determining gene in *X. laevis. *In *R. rugosa* the expression of steroidogenic genes, such as *cyp11a1*, *star*, *hsd3b*, *cyp17*, *hsd17*β**, and *cyp19* and gene encoding 5-*α*-reductase, starts in the indifferent gonads of male and female tadpoles prior to sex determination [11].

**Figure 1:**
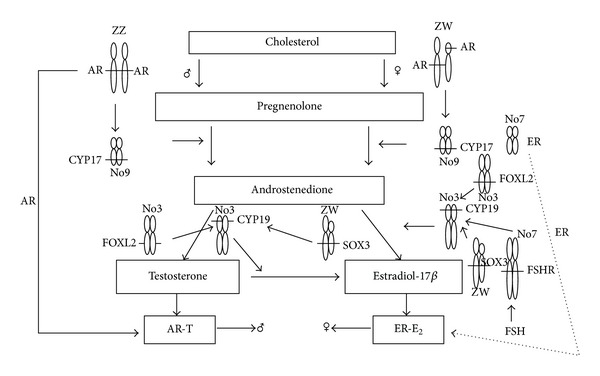
This schema presents sex determination by steroid hormones and some SD-genes in *Rana rugosa*. Testosterone and estradiol-17*β* are produced in the undifferentiated gonads of males and females, respectively. Sex chromosomes (Z, W) and autosomes (3, 9, 7) were denoted by letters and the numbers, respectively. AR-T and ER-E_2_ indicate the complexes of steroid receptor. Localization of genes on chromosomes marked lateral line (adopted from [10, 11]).

At present time, the sex-determining genes in some species at amphibians are not known. However, apart from the sex-determining genes, genes involved in gonadal differentiation may be conserved in all classes of vertebrates. This is supported by the fact that genes such as *foxl2*, *dmrt1*, *wt1*, *sox9*, *sf1*, *cyp19*, and *dax1* are evolutionary conserved genes from fish to mammals. The exogenous steroid hormones can determine the phenotypic sex of many species of amphibians. For example, XX female-to-male sex reversal in *R. rugosa *can be induced by testosterone although XX-females do not carry a male-determining gene on the X chromosome. Thus, a sex-determining gene is not actually necessary for sex determination in amphibians. In the other words, the steroid hormones could be the key factor for sex determination in amphibians. A male sex-determining gene, if it exists, probably supports steroid hormones to direct indifferent gonads to a testis by inhibiting *cyp19* transcription for ovarian formation. At present, factors for upregulation of *cyp17* in the indifferent gonad of *R. rugosa *remain to be identified (Figure 1) [10, 11].

## 2. Estrogens and Nonmammalian Vertebrate Sex Determination

Estrogen is both necessary and sufficient to drive ovarian development in many nonmammalian vertebrates (Table 1) [12]. Moreover there is the actual material about this hormone is able influence not only the differentiation of sex but also the appearance of specific sex gonads (sex determination) (Table 2) [13]. However, the role of estrogen in the mammalian gonad is less clear. Mouse ovarian development can proceed in the absence of estrogen signaling, but granulosa cell fate cannot be maintained. Estrogen receptor expression is conserved in the indifferent gonad of all mammals and many species also express the *cyp19* gene that encodes aromatase in the early ovary [13].

Furthermore, the estrogen is sufficient to drive ovarian development of the indifferent gonad in marsupial mammals. Estrogen treatment in alligators and turtles at the male-producing temperature induced ovarian transformation, including proliferation and entry of germ cells in the cortex into meiosis, and at the same time, degeneration of sex cords in the medullary region. Estrogen appears to promote the ovarian fate by stimulating the expansion of the cortex while inhibiting the maintenance of sex cords [13]. Animals utilizing TSD are susceptible to increases in temperature, as well as exposure to chemicals, such as synthetic estrogens. These factors have the potential to skew sex ratios. It is possible that controlling sex at the chromosomal level evolved as a protective mechanism in order to shield embryos from the changing environment [12, 13].

Birds and marsupials are unique because their sex is determined by classical GSD mechanism although the embryo remains sensitive to the effects of estrogen. It is especially so for the chicken *Gallus gallus* and tammar wallaby *Macropus eugenii*, the most characterized models for avian and marsupial species, respectively. Although birds diverged from reptiles approximately 245 million years ago, the ovary-determining action of estrogen remains present in birds with the evolution of a genetic mechanism for sex determination. This phenomenon is also found in marsupials, the close relatives to the eutherian mammals. Most marsupials, such as the tammar wallaby, are born underdeveloped with a mixture of fetal and neonatal characteristics. Gonadal development in the marsupial is similar to that of mice and humans except that the development of marsupial fetuses occurs outside of the uterus. Marsupial gonads are sexually indifferent at birth, and gonadal differentiation commences immediately after birth. By the 7th postnatal day, the ovarian differentiation has progressed to the point where the gonad is morphologically distinguishable. In the tammar wallaby, unlike birds and reptiles, the ovary does not produce estrogen at the time of gonadal differentiation. In fact, the ovarian steroid production does not commence until almost 200 days after birth. Based on this fact, it appears that estrogen is not necessary for the differentiation of the ovary as it is in birds and reptiles. However, the administration of estrogen to wallaby male embryos immediately after birth can induce complete ovarian development similar to reptiles and birds. The ability of estrogen to feminize the male gonad in the marsupial suggests that estrogen can override the genetic components derived from the XY mechanism. It is possible to induce chicken sex reversal by using inhibitor of aromatase or the analogs of estrogen (Table 2) [13–16].

Mammals evolved from lower vertebrates approximately 80 million years ago. Accompanying that recent evolution was a new form of sex determination, GSD that utilizes only chromosomal composition to determine sex. Unlike birds and marsupials that use GSD but remain sensitive to steroids, the eutherian mammals evolved a mechanism whereby sex determination is completely resistant to steroids. While it appears that estrogen has no impact on primary sex determination in the developing eutherian embryo, a different situation arises after birth. In the adult mammalian female, estrogen plays a vital role in maintaining the ovary. In the aromatase knockout mouse, testicular cell types and structures arose in postpubertal ovaries. A similar phenomenon was observed in mice lacking both estrogen receptors *α* and *β* (ER*αβ*KO), where Sertoli cells and seminiferous-like structures appeared in the ovaries after puberty (Table 2). These observations bring about an intriguing hypothesis that the primitive estrogen-induced mechanism of ovary development remains present in eutherian females [13].

According to Pask [12], in the presence of estrogen, key male differentiation genes fail to be upregulated in the XY gonad and instead key ovary-promoting genes are upregulated leading to ovarian development. Estrogen appears to trigger sex reversal through the exclusion of *sox9* from entering the nucleus in the somatic cells of the developing gonad of nonmammalian vertebrate. In the absence of nuclear *sox9*, Sertoli cell development cannot be initiated and the somatic cells follow a granulosa cell fate. A conserved role for estrogen-mediating *sox9* action is consistent with several observations in mammals. In mice, *sox9* is able to autoregulate by binding to its own promoter. Activated estrogen receptor complexes can also move into the nucleus and along with *foxl2*, suppress *sox9* transcription by directly binding to the *sox9* enhancer, TESCO.

## 3. Sex-Determining Genes in Vertebrates

Although the molecular mechanisms underlying many developmental events are conserved across vertebrate taxa, the lability at the top of the sex-determining (SD) cascade has been evident from the fact that four master SD genes have been identified: *sry* (mammalian), *dmrt1 *(chicken), *dmy* (medaka), and *DM-W* (*Xenopus laevis*) [8, 18]. Recently four novel candidates for vertebrate SD genes were reported, all of them are in fishes. These include *amhy* in the Patagonian pejerrey, *gsdf* in *Oryzias luzonensis, amhr2* in fugu, and *sdY* in rainbow trout (*Salmo gairdneri*) (Table 3) [18]. This large-scale natural experiment provides a resource that geneticists can use to search genetic variants of sex determination control. Accumulation of knowledge on such variants will allow us to distinguish conserved and diversified SD pathways among vertebrates and lead us into a deeper understanding of the vertebrate SD cascade. Given the evidence from all the fish species mentioned, together with previous studies of other nonmammalian species, it seems reasonable to imagine that many other teleosts, reptiles and amphibians also have experienced a turnover of sex chromosomes [8].

The comparison of new and old SD genes indicates that vertebrate sex-determining cascades are not as conserved as once thought. In eutherian mammals, *sry* is a recently evolved key Y-linked testis determinant. *Sry* is absent from the genome outside therian mammals (marsupials and placentals). In birds and lower vertebrates, there is a pervasive role for DM domain of genes in gonadal sex differentiation, and it is considered that these genes have an ancient association with sex. In mammals, the current evidence favors the idea that *sry* acts with the orphan nuclear receptor Sf1 to activate *sox9* expression in the developing XY gonad. *Sry* is turned off by *sox9* which then maintains its own expression. However, in the chicken embryo, *dmrt1* expression precedes that of *sox9* by at least 2 days (the 4th embryonic day versus the 6th day), implying that other intervening genes are involved. Interestingly, in the medaka fish, gene *sox9b* (the orthologue of tetrapod *sox9*) is not involved in testis determination, but has a function in germ cells. In lower vertebrates, *sry* is also absent and other triggers must exist. Given the different, independent origins of sex chromosomes among birds, reptiles, and amniotes, a variety of different testis-determining triggers are likely. In birds, testis development requires presumably the conserved Z-linked *dmrt1 *gene. Yet, *dmrt1* is not sex-linked in various reptiles, pointing to another factor being involved. In mammals, *sox9* activates expression of the *fgf9* and genes encoding prostaglandin D synthase (*pdgs*) [7, 8, 17].

**Figure 2:**
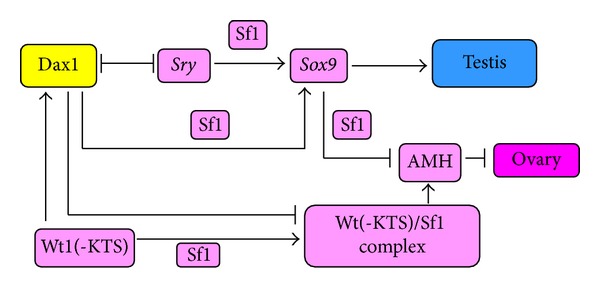
The role AMH in repressing of Müllerian duck differentiation. The Wolffian duck has to be maintained and stimulated to differentiate into the male tract and accessory organ. Then, the Müllerian duct system has to regress, due to action AMH secreted by Sertoli cells. *Sox9* and *Sf1* are both involved in the expression of the *AMH* gene as a result of their respective binding to the promoter and in part because of their ability to interact with each other (adopted from [17]).

In the mammalian gonad, a key role for *sox9* during testicular development is the activation of Amh (anti-Müllerian hormone) (Figure 2, [17]).

**Figure 3:**
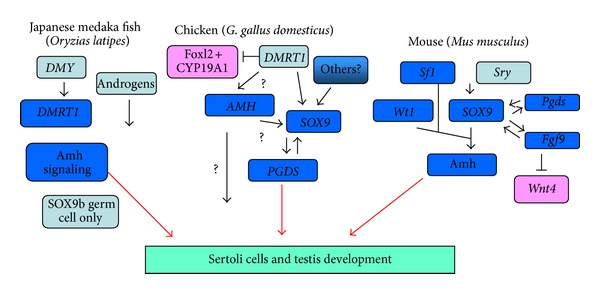
Genetic determination of testes in Japanese medaka fish, chicken, and mouse. Somatic gene *sox9* was involved in the control of testes from fish. AMH signaling pathway is a very conservative element of testes, along with other major regulators, appeared in other species (adopted from [4]).

In the chicken embryo, *amh* precedes expression of *sox9*, and the gene is expressed in both males and females. Hence, *sox9* does not activate *amh* in the avian system although it might upregulate it. The activator of expression of *amh* in the chicken and other vertebrates is unclear, but is likely to involve the orphan nuclear receptor SF1 which is expressed in male gonads (ZZ) compared to females (ZW) suggesting that a dosed Z-linked gene such as *dmrt1 *may activate its expression in avians. *Amh* may participate in testis determination by blocking estrogen synthesis, namely by repressing expression of the aromatase encoding gene *cyp19a1* (Figure 3) [4].

**Figure 4:**
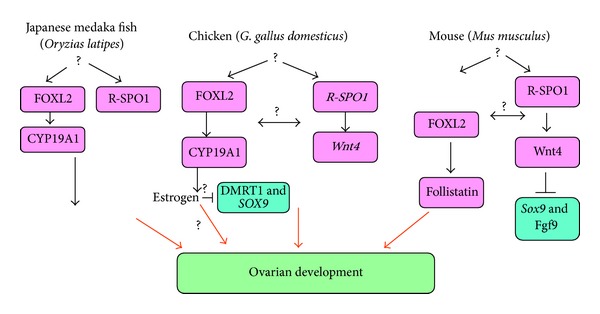
Genetic determination of the ovaries of Japanese medaka fish, chicken, and mouse. Genes such as *foxl2* and *r-spo1* are conserved. *Cyp19a1* plays a more important role in nonmammals (adopted from [4]).

The key ovary determinant in mammals has not yet been defined, but the canonical *β*-catenin signaling pathway is required for ovarian morphogenesis [4]. The *β*-catenin signaling pathway appears to be conserved in the other vertebrates, including fishes, reptiles with TSD, and chickens. In these cases, R-Spo1, Wnt4, and/or *β*-catenin show female upregulation [4, 19]. This female pathway appears to be deeply conserved among vertebrates. A major difference between mammal and nonmammalian vertebrates is the requirement of estrogen for ovarian differentiation in the latter [4]. In eutherian mammals, the embryonic gonads are resistant to sex steroid effects although estrogen is required to maintain the postnatal ovary. The position of *foxl2 *in the ovarian pathway appears to vary among the major groups. In the chicken embryo, *foxl2 *expression from 5.5th embryonic day is one of the earliest known markers of ovarian development. *Foxl2 *activates the *cyp19a1* 5′-regulatory region in the tilapia fish and in mammals. A major question relating to ovarian development is how the FOXL2 and R-SPO1/Wnt4 pathways interact to coordinate ovarian development. The two pathways appear to be independent in goat and in chicken embryos; FOXL2 and R-SPO1 localize to different ovarian compartments (medulla and cortex, resp.) (Figure 4) [4].

Accumulation of knowledge on recent variants of sex determination will allow us to distinguish conserved and diversified SD pathways among vertebrates and lead us into a deeper understanding of the vertebrate SD cascade. For example, it may be worth reconsidering the role of AMH signaling in gonadal sex determination even in mammals based on the results of previous fish studies [7, 8]. Until 2011, all four vertebrate master SD genes (or strong candidates) were known to code for transcription factors which could have been construed as evidence that gonadal sex determination in vertebrates is always triggered by transcription factors. However, the three novel candidates for the master SD genes in the Patagonian pejerrey, *Oryzias o. luzonensis*, and fugu code for growth factors or one of their receptors. Thus, these findings suggest alternative mechanisms of genotypic sex determination, in which the main trigger is not constrained to be a transcription factor [8, 20].

A growing body of evidence reveals the importance of epigenetic regulatory mechanisms, such as DNA-methylation, histone modifications, and the role of noncoding RNAs in controlling sex determination and gonadogenesis. Chicken Z-linking MHM region and specific sites of hypermethylated inside chicken *cyp19*, involvement of methylated lysine 9 of histone H3 (H3K9me) and heterochromatin protein 1 (HP1) in epigenetic modifications in the phenomenon of imprinting, and the presence conserved PHF7 protein from insects to mammals that exhibits male-specific expression in the germline can be mentioned as examples [14, 15, 21].

## 4. Summary

Different groups of vertebrates discovered the changes of all stages of the formation of sex characteristic of mammals: the chromosomal sex predetermination associated with the formation of the XX or XY zygote at fertilization, formation of testes or ovary (sex determination), and the full development of the respective gonads, associated features (sex differentiation). So much for the birds and Lepidoptera described heterogametic females and merging Z and W chromosomes at fertilization. For mammals, birds, amphibians, and fish, are revealed sex determining genes. Only some of them belong to the families of such as *SOX* genes and DM-containing genes, which are transcription factors similarly controlled the sex determination. Other fish genes were described (*Gsdf* in *Oryzias luzonensis*, *amhr2* in *Takifugu*, *amhy* in the Patagonian pejerrey, *sdY* in *Oncorhynchus mykiss*). The genes belong to completely different families encoding growth factors or receptors and they can influence directly on the proliferation of germ cells. It is possible to talk about specific plasticity of sex determination of fish. In amphibians, sex-determining genes virtually replaced steroids ones. The role of steroids in sex determination is clearly weaker in the evolutionary chain of amphibians, reptiles, birds, mammals, and marsupials. Around this scheme, the transition is implemented from pure TSD mechanism to pure GSD one.

A general feature of vertebrate gonadal sex-determining pathways is that master switches appear to have been added at the top of the hierarchy, with more conserved core genes appearing downstream. Genes *amh *and *sox9* in males are conserved across groups, but the upstream regulator differs (*sry *in mammals and DM domain genes in nonmammals). With respect to the ovarian development, the R-SPO1/Wnt4 and FOXL2 pathways are conserved from fishes to birds and mammals. However, the genetic networks are in fact more complex than this simple scenario. It would appear that the testis pathway is more changeable than the ovarian pathway although this cannot be confirmed in the absence of master ovary factors. For example, in male embryos, the Sertoli cell progenitors in the chicken apparently have a different developmental origin from those of the mouse. Differences between species must logically involve differences in gene expression, and this is reflected in the past—the embryonic gonads of vertebrates have been considered highly conserved in structure. While this is generally true, it is clear that the molecular pathways underlying this commonality of structure are actually quite plastic. Functional analyses are now possible in most groups, such as fishes and chicken, in addition to well-established strategies in mouse. Another area worthy of investigation is exactly how novel genes are incorporated to the top of the pathway and determining the evolutionary pressures that cause the relative shuffling of genes in the male and female pathways. These efforts will broaden our understanding of vertebrate sex determination and how it has evolved [4, 8, 16].

## Figures and Tables

**Table 1:** The role of estrogen in sex determination of vertebrates and phylogenetic distance (adopted from [12]).

Group of vertebrata	Distance from mammals (million years ago, MYR)	Influence of estrogen on sex determination
Nonmammalian vertebrata		

Pisces	450–530	+
Amphibia	300	+
Reptilia	290	+
Aves	216–199	+

Theria		

Monotremata	180	+
Marsupialia	160	+
Eutheria (Mammalia)	0	—

**Table 2:** Mechanisms of sex determination and experimental sex reversal in vertebrates (adopted from [13]).

Mechanism of sex determination	Species	Effectors	Experimental evidence of sex reversal
TSD (t°C)	*American alligator and Red-eared slider turtle *	Aromatase or Estradiol (E_2_)	F-M: administration of an aromatase inhibitor introduced at F-producing temperatures; M-F: E_2_ administration to eggs incubated at male-producing temperatures

GSD (XX/XY; ZZ/ZW)	*Oryzias latipes, Bufo bufo*,* and Xenopus laevis *	Androgene or E_2_	—

GSD (ZZ/ZW)	*Gallus gallus *	Aromatase or Estradiol (E_2_)	F-M: in ovo administration of an aromatase inhibitor to ZW animals; M-F: in ovo administration of E_2_ to ZZ individuals

GSD (XX/XY)	*Macropus eugenii and Mus musculus *	*Sry *	F-M: treatment of XX individuals with M$\ddot{\text{u}}$llerian inhibiting substance (MIS) to cause germ loss, addition of *Sry* gene in XX embryos; loss of E_2_ through ER*αβ*KO causes transdifferentiation of the ovary to testis-like structures in the adult; M-F: inactivation of *Sry* gene in XY embryos, E_2_ administration to XY

Here: F: female, M: male, E_2_: estrogene, X, Y, Z, W: sex chromosomes.

**Table 3:** The known sex-determining genes in vertebrate (adopted from [18]).

Species	The known sex-determing genes	The main peculiarities of the genes
Mammals		

*Homo sapiens, Mus musculus, *and so forth*. *	*Sry *	Transcription factor, upregulator of *Sox9*; and testis-determining gene.

Birds		

*Gallus gallus domesticus, *and so forth.	*Dmrt1 *	Transcription factor, upregulator of *Sox9, *DM domain gene; and testis-determining gene.

Amphibia		

*Xenopus laevis *	*DM-W *	Transcription factor, DM domain gene, and ovary-determining gene.

Fishes		

*Oryzias latipes *	*Dmy *	Transcription factor, DM domain gene, and testis determining gene.
*Oryzias luzonensis *	*Gsdf *	Secretory protein belonging to the TGF-*β* superfamily.
*Takifugu *	*Amhr2 *	Receptor for Amh.
Patagonian Pejerrey	*Amhy *	The Amh protein has been implicated in the regulation of germ cell proliferation and spermatogenesis.
*Oncorhynchus mykiss *	*SdY *	A novel protein that displays sequence homology with carboxy-terminal domain of interferon regulatory factor 9 (Irf9).

## Abbreviations

GSD: Genetic sex determination
ESD: External sex determination
TSD: Temperature sex determination
X, Y, Z, W: Sex chromosomes
SD-gene: Sex determination gene
ER: Estrogen receptor
AR: Androgen receptor
AMH: Anti-Müllerian hormone
T: Testosterone
E_2_: Estradiol
MYR: Million years ago
F: Female
M: Male.
